# Evaluating the accuracy of ICD-10 codes for syncytial respiratory virus diagnosis in hospitalized patients: A record-linkage study (2022–2023)

**DOI:** 10.1371/journal.pone.0319436

**Published:** 2025-03-28

**Authors:** Ana Luiza Bierrenbach, Olivia Tavares Ranzani

**Affiliations:** 1 Precision Data, São Paulo, São Paulo, Brazil; 2 Instituto de Ensino e Pesquisa, Hospital Sírio-Libanês, São Paulo, São Paulo, Brazil; 3 Santa Casa de Misericórdia de Mogi Mirim, Mogi Mirim, São Paulo, Brazil; 4 Olita Pesquisas Científicas Ltda, Mogi Mirim, São Paulo, Brazil; Universitair Kinderziekenhuis Koningin Fabiola: Hopital Universitaire des Enfants Reine Fabiola, BELGIUM

## Abstract

**Background:**

Respiratory Syncytial Virus (RSV) is a leading cause of severe respiratory infections in young children and older adults. Accurate RSV surveillance is essential to understanding its disease burden and evaluating vaccine impact.

**Methods:**

We assessed the accuracy of ICD-10 coding for RSV hospitalizations in the Brazilian Hospital Information System (SIH) by linking it with the Severe Acute Respiratory Syndrome (SIVEP) notification system (2022–2023). Laboratory-confirmed RSV-positive and RSV-negative cases in SIVEP were used as the reference standard. Sensitivity and specificity were evaluated for ICD-10 definitions (RSV, RSV +  J21 [Acute Bronchiolitis], RSV +  Acute Respiratory Infection) overall and by age group (under 1 year, under 5 years, and over 60 years). The top 10 diagnoses of RSV-positive patients were also analyzed by age group.

**Results:**

Among 15,169 RSV-positive patients linked to an SIH record, 73.0% were under 12 months old, 20.8% were 1-5 years old, 3.7% were 5-59 years old, and 2.5% were 60 + years. Acute bronchiolitis was the most common diagnosis overall (43.5%), particularly in infants (53.5%). In older adults, pneumonia due to unspecified microorganisms was most frequent (24.6%). Sensitivity improved with broader case definitions, such as, RSV +  Acute Respiratory Infection (66.7%, 95%CI: 65.8–67.6 in infants; 23%, 95%CI: 18.9–27.6 in older adults). Specificity was higher in older adults (83.8%, 95%CI: 83.5–84.1) than in infants (45.1%, 95%CI: 44.4–45.6). Additionally, 40,701 RSV-positive notified cases lacked RSV-coded diagnoses in SIH.

**Conclusion:**

Our study highlights the discrepancy between RSV-positive cases identified in SIVEP and those coded in the SIH database, reflecting limitations in ICD-10 coding, particularly in the older population. Reliance on symptomatic coding rather than confirmed diagnoses contributes to this issue. Accurate RSV identification is crucial, especially with new vaccines available. Improved diagnostic coding is essential for effective RSV surveillance and evaluating vaccine impact.

## Introduction

Respiratory Syncytial Virus (RSV) is increasingly recognized as a significant cause of severe respiratory infections, particularly in young children and more recently among older adults [1]. Despite this, the accurate identification and documentation of RSV infections in healthcare settings remain challenging, largely due to overlapping clinical presentations with other respiratory viruses, such as influenza and COVID-19 [2–4]. The International Classification of Diseases, 10th Revision (ICD-10), is routinely used to code diagnoses in healthcare databases, but its reliability in accurately capturing acute respiratory infections has been investigated more extensively for influenza than for RSV [5].

While studies estimating the validity of ICD-10 codes for identifying laboratory-confirmed influenza have produced mixed results [6–11], the accuracy of RSV-specific ICD-10 codes in identifying true RSV infections has received relatively little attention. To our knowledge, only a couple studies have assessed the accuracy of these codes. Pisesky et al. reported high sensitivity (97.9%, 95%CI: 95.5%-99.2%) and specificity (99.6%, 95%CI: 98.2%-99.8%) of RSV-specific ICD-10 codes for identifying hospitalized RSV cases in children under 3 years of age at a pediatric reference hospital in Ontario, Canada. However, the sensitivity for identifying RSV cases in the emergency care setting was lower (49.5%, 95%CI: 43.7%-55.3%), with the specificity remaining high (99.6%, 95%CI: 99.5%-99.8%) [12]. In contrast, Cai et al. found poor sensitivity (6%, 95%CI: 3%-12%) and high specificity (99.8%, 95%CI: 99.6%-99.9%) for RSV codes in the general population attending 48 primary or secondary care practices in Germany. When considering only children under five years of age, the sensitivity was somewhat higher (14%, 95%CI: 6%-20%), while specificity remained high (99.6%, 95%CI: 98%-99.9%) [13]. Similarly, Egeskov-Cavling et al. evaluated national data from Denmark (2015–2018), linking patients aged 18 years or older with respiratory tract infection-coded hospitalizations to RSV test results. They reported an overall sensitivity of RSV-coded hospitalizations of 42.4% (95%CI: 39.3%–45.6%) [14]. These findings suggest that RSV may be underdiagnosed and undercoded, particularly in populations outside of pediatric care. Other reasons, such as accuracy improving during RSV seasonality and varying depending on the type of healthcare facility reporting, have also been noted in these studies.

In Brazil, the epidemiological profile of RSV varies by region and population. Seasonality differs across the country, with southern Brazil experiencing RSV peaks during colder months and central and northern regions during the rainy season, which varies significantly in timing and intensity across these areas [15–19]. Young children, particularly those under 12 months, bear the highest burden of RSV-related hospitalizations, while older adults are also significantly affected, often presenting with complications such as pneumonia [20,21]. Recently, two RSV vaccines have been approved by Brazil’s National Health Surveillance Agency (Anvisa) and are available in the private healthcare sector. However, these vaccines are not yet part of Brazil’s National Immunization Program (PNI) [21]. These regional and age-related differences in RSV burden, alongside the availability of new vaccines in the private sector, highlight the importance of accurate surveillance and coding practices to monitor disease trends and guide public health strategies effectively.

The need for accurate identification of RSV infections has become more urgent with the recent approval of the first RSV vaccines and the fact that others are in the pipeline [22–24]. The planning of future RSV vaccination strategies and the evaluation of the impact of these vaccines will rely heavily on accurate and timely RSV epidemiological data, as well as long-term observation of RSV seasonality through surveillance systems. Therefore, understanding the limitations and accuracy of current diagnostic coding practices is crucial.

This study aims to assess the sensitivity and specificity of ICD-10 codes in identifying RSV infections across different age groups in Brazil. We focus on data from 2022 and 2023, reflecting the COVID-19 post-pandemic scenario when the use of viral PCR testing for acute respiratory infections (ARI) has significantly increased [25]. The inclusion of this recent data is particularly important as it captures the evolving landscape of respiratory diagnostics and disease burden in the context of heightened awareness and testing capabilities introduced during the COVID-19 pandemic.

By linking data from the national hospitalization database with the Severe Acute Respiratory Syndrome (SARS) notification database, we seek to determine the frequency and accuracy of RSV coding. Our hypothesis is that RSV is frequently underdiagnosed and undercoded, particularly among older adults, which could lead to an underestimation of the disease burden in this vulnerable population. The findings of this study are expected to inform strategies for improving clinical documentation, enhancing public health surveillance, and supporting the effective implementation of RSV vaccination programs as these vaccines become available.

## Methodology

This study evaluates the accuracy of ICD-10 coding for RSV hospitalizations in the Brazilian Hospital Information System (SIH) by linking it with the national SARS notification system (SIVEP) from January 2022 to December 2023. The goal is to validate RSV-related hospitalizations in SIH, comparing ICD-10 coding against RSV-positive and RSV-negative cases recorded in SIVEP, used as the reference standard.

The SIH was initially created as a financial tool to reimburse hospitals within Brazil’s public healthcare system (SUS). It covers approximately 75% of all hospitalizations in Brazil [26], excluding those in private or non-affiliated hospitals, which account for the remaining 25% of the population [27]. Hospitals submit monthly reports containing diagnostic and procedural data. Despite its primary administrative and financial purpose, the SIH database has become increasingly important for epidemiological studies, public health surveillance, and assessments of healthcare performance.

The SIH coding process follows Ministry of Health guidelines based on the standard international version of the ICD-10 provided by the WHO. Coding is typically performed by hospital administrative staff using documentation such as discharge summaries, medical records and diagnostic tests. The system focuses on reimbursement-related codes, which can prioritize resource utilization over detailed clinical diagnoses, leading to inconsistencies. Challenges such as insufficient training, high staff turnover, and low pay further impact coding quality. While municipal and regional health secretariats audit the process to ensure compliance, systemic issues like workload and institutional practices continue to affect accuracy [28,29]. The Ministry of Health provides coding guidelines through resources like the ‘Manual Técnico Operacional do Sistema de Informações Hospitalares do SUS,’ available on the DATASUS website [30].

SIVEP, initially implemented in 2000 for sentinel reporting of flu-like syndromes, was expanded nationwide in 2009 during the H1N1 Influenza A pandemic to include notifications for Severe Acute Respiratory Syndrome (SARS) [31]. In 2020, the system was further adapted to include COVID-19 cases. SIVEP mandates the reporting of any hospitalized case or death due to SARS within 24 hours, regardless of hospitalization status or death, by registered healthcare units. The case definition for a flu-like syndrome requires the presence of at least two symptoms, including fever, chills, sore throat, headache, cough, or loss of taste/smell. Severe cases (SARS) are defined by the presence of respiratory distress, persistent chest pressure, oxygen saturation below 95%, or cyanosis in an individual with a flu-like syndrome. Both public and private healthcare facilities are required to report SARS cases. All SARS hospitalizations should be reported as well as SARS deaths, independently of hospitalization status. Notification forms are completed by staff responsible for epidemiological surveillance at each registered unit.

The SIVEP system is primarily completed by healthcare professionals, with hospital nurses playing a central role in entering data for Severe Acute Respiratory Syndrome (SRAG) cases. They are responsible for recording patient information, clinical details, and laboratory results. The Ministry of Health provides guidance through manuals like the ‘Guia Rápido SIVEP-Gripe (Quick guide to SIVEP-Gripe)’ and the ‘Guia de Vigilância Integrada da COVID-19, Influenza e Outros Vírus Respiratórios (Integrated Surveillance Guide for COVID-19, Influenza, and Other Respiratory Viruses)’, which outline standardized procedures for data entry and case management [32,33]. However, variability in training, user experience, and workload can affect data consistency and accuracy, similar to challenges observed in the SIH system.

Data from both the SIH and SIVEP databases were publicly accessed via the SUS Department of Informatics website, with patient confidentiality ensured as no identifying information was included. Both datasets were cleaned to remove duplicates across all variables, followed by the exclusion of duplicates specifically within the variables used for the linkage procedures. Non-hospitalized cases were also excluded from SIVEP. A deterministic linkage method was used, requiring exact agreement on all linkage variables—admission date, discharge date, birthdate, sex, municipality of residence, and hospital code—for records to be matched between the two datasets. The workflow and a Venn diagram of the linkage process are provided in the results section.

In SIVEP, RSV cases were defined as those with a final classification marked as “SARS by another respiratory virus” in the final classification (CLASSI_FIN) variable, combined with a positive result in either the rapid antigen test (AN_VSR) or polymerase chain reaction test (PCR_VSR) variables. In SIH, RSV hospitalizations were identified using specific ICD-10 codes grouped into 3 case definitions, as outlined in Table 1. These extended definitions are similar to those proposed by Cai et al. [13]

**Table 1:** Case definitions for identifying RSV cases in the SIH based on ICD-10 coding.

Codes	Code names
1. RSV	
B97.4	RSV as the cause of diseases classified to other chapters
J12.1	RSV pneumonia
J20.5	Acute bronchitis due to RSV
J21.0	Acute bronchiolitis due to RSV
2. RSV + Acute bronchiolitis	
	All RSV specific codes, plus:
J21^*^	Acute bronchiolitis
J21.8	Acute bronchiolitis due to other specified organisms
J21.9	Acute bronchiolitis, unspecified
3. RSV + Acute Respiratory Infection	
	All RSV + Acute bronchiolitis codes, plus:
B34.9	Viral infection, unspecified
J06^*^	Acute upper respiratory infections of multiple and unspecified sites
J06.0	Acute laryngopharyngitis
J06.8	Other acute upper respiratory infections of multiple sites
J06.9	Acute upper respiratory infection, unspecified
J11^*^	Influenza, virus not identified
J11.0	Influenza with pneumonia, virus not identified
J11.1	Influenza with other respiratory manifestations, virus not identified
J11.8	Influenza with other manifestations, virus not identified
J12^*^	Viral pneumonia, not elsewhere classified
J12.8	Other viral pneumonia
J12.9	Viral pneumonia, unspecified
J18^*^	Pneumonia, organism unspecified
J18.0	Bronchopneumonia, unspecified
J18.8	Other pneumonia, organism unspecified
J18.9	Pneumonia, unspecified
J20^*^	Acute bronchitis
J20.8	Acute bronchitis due to other specified organisms
J20.9	Acute bronchitis, unspecified
J22^*^	Unspecified acute lower respiratory infection

Codes marked with an asterisk (*) refer to categories recorded without specifying further subcategories.

The accuracy of each ICD-10 case definition (RSV, RSV +  J21, RSV +  ARI) was assessed using sensitivity, specificity, and the area under the ROC curve (AUC), with the AUC representing the average of sensitivity and specificity. For each definition, we calculated 95% confidence intervals based on the binomial distribution. Accuracy metrics were evaluated for the entire population and for specific age groups: under 1 year, under 5 years, and over 60 years. Additionally, we present the top 10 clinical diagnoses of hospitalized RSV-positive patients across the different age groups.

Data management, analysis, and the record linkage process were performed using STATA 17 (StataCorp LLC, College Station, TX, USA).

## Results

**Fig 1:**
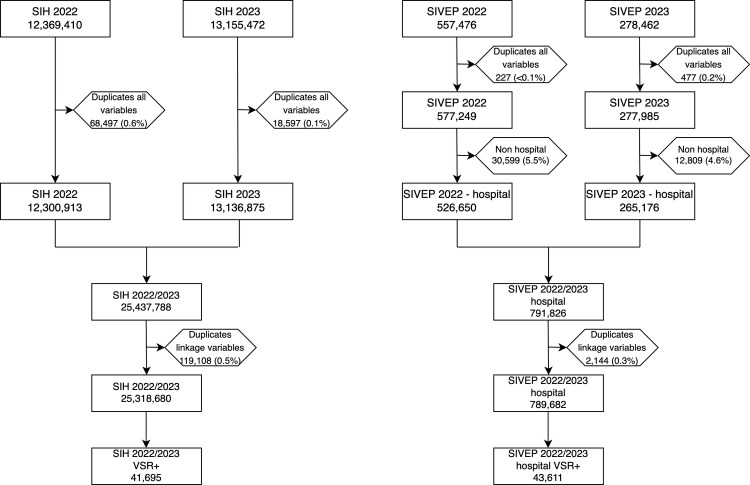
Data cleaning and processing workflow for the SIH and SIVEP datasets (2022-2023).

Fig 1 presents the workflow detailing the data cleaning and processing steps for the 2022 and 2023 national hospitalization (SIH) and national notification (SIVEP) datasets. In the SIH dataset, 25,437,788 records were initially retrieved after removing duplicates across all variables. An additional 119,108 records (0.5%) were then eliminated as they were duplicates based on a restricted set of variables intended for linkage between the SIH and SIVEP datasets, including date of admission, date of discharge, date of birth, sex, municipality of residence code, and hospital unit code, resulting in a final dataset of 25,318,680 records. Among these, 41,695 records (0.2%) were identified as RSV-positive, with the majority (95.3%) having a primary diagnosis of J21.0 (RSV acute bronchiolitis), followed by J12.1 (RSV pneumonia) at 4.5%, J20.5 (RSV acute bronchitis) at 0.2% and B97.4 (RSV as the cause of diseases classified elsewhere) at less than 0.1%. No RSV codes were found in the secondary diagnosis fields.

In the SIVEP dataset, 791,826 records were retrieved after removing duplicates across all variables and excluding non-hospital records, which accounted for only 5.1% of the total. Subsequently, an additional 2,144 duplicates (0.3%) based on the linkage variables were eliminated, resulting in a final dataset of 789,682 records. Among these, 43,611 records (5.5%) were identified as RSV-positive, with 84.3% diagnosed through PCR testing and 15.7% through rapid antigen testing.

**Fig 2:**
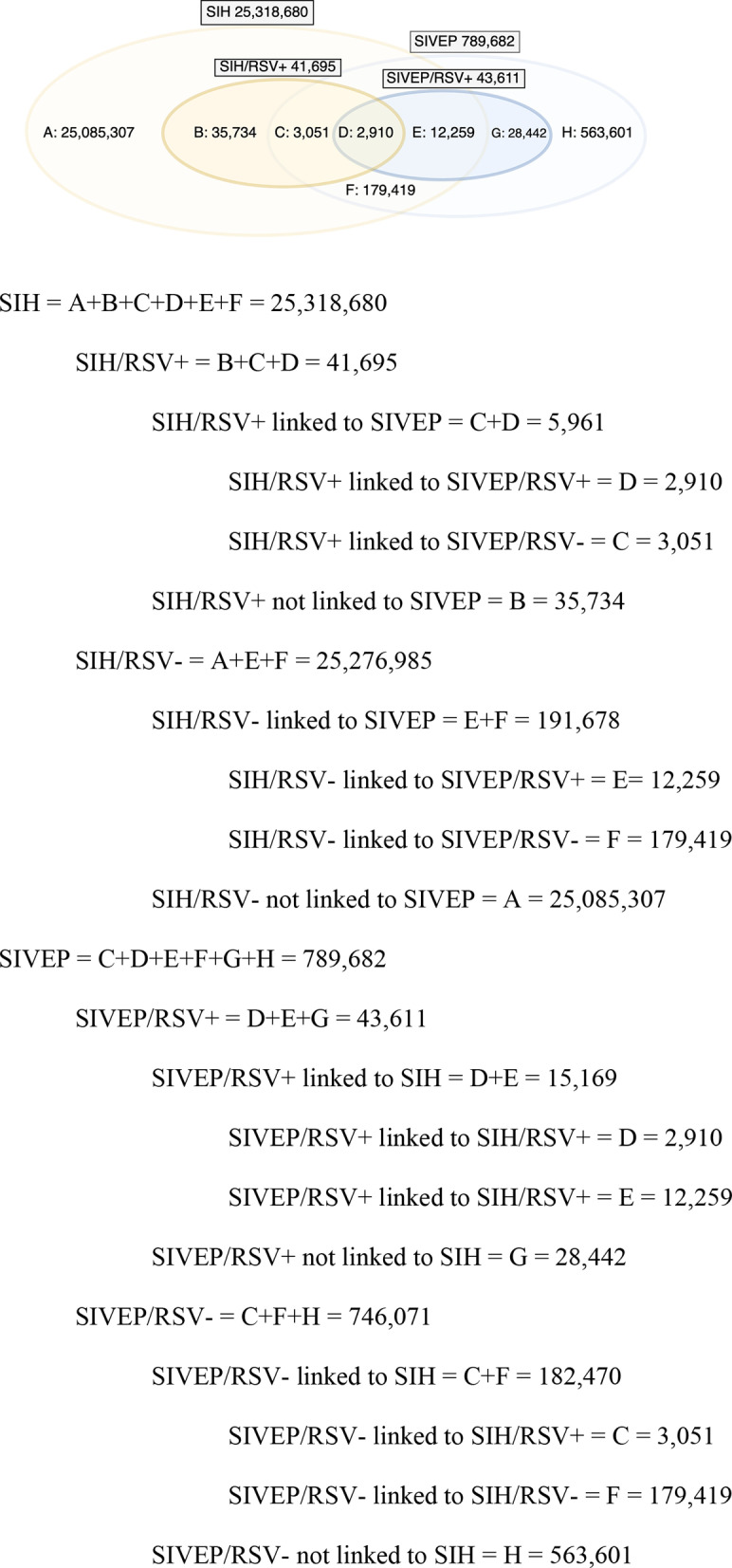
Venn diagram of linkage between SIH and SIVEP datasets (2022–2023).

Fig 2 shows the Venn diagram of the linkage process between the 25,318,680 records from SIH and the 789,682 records from SIVEP. From the SIH perspective, the linkage proportion is approximately 0.8%, while from the SIVEP perspective, it is 25%. Among the 41,695 RSV-positive records in the SIH dataset, 5,961 (14.3%) were successfully linked to a corresponding record in the SIVEP dataset. Conversely, of the 43,611 RSV-positive records in SIVEP, 15,169 (34.8%) were linked to an SIH record.

Table 2 presents the top 10 clinical diagnoses of these 15,169 SIVEP RSV-positive patients with corresponding SIH records. Among these patients, 73.0% were under 12 months old, 20.8% were 1-5 years old, 3.7% were 5-59 years old, and 2.5% were 60 years or older. In the overall population, acute bronchiolitis was the most common diagnosis (43.5%), followed by pneumonia due to unspecified microorganisms (18.1%) and bacterial pneumonia, unspecified (7.4%). In children under 1-year, acute bronchiolitis was even more prevalent (53.5%), with pneumonia due to unspecified microorganisms (15.2%) and respiratory failure (6.4%) also being frequent. Similarly, in children under 5 years, acute bronchiolitis remained the leading diagnosis (46.2%), followed by pneumonia due to unspecified microorganisms (17.8%) and bacterial pneumonia, unspecified (7.3%). In contrast, the population over 60 years displayed a different pattern, where pneumonia due to unspecified microorganisms was the most frequent diagnosis (24.6%), followed by other chronic obstructive pulmonary diseases (11.4%) and heart failure (8.5%). Unlike the younger age groups, acute bronchiolitis was not among the top diagnoses in this older population.

**Table 2:** Top 10 clinical diagnoses of hospitalized RSV-positive patients across different age groups.

Diagnosis	ICD-10	Freq.	Percent	Cum.
Whole population				
Acute bronchiolitis	J21	6,601	43.5	43.5
Pneumonia due to unspecified microorganism	J18	2,750	18.1	61.6
Bacterial pneumonia, unspecified	J15	1,125	7.4	69.1
Respiratory failure, not elsewhere classified (NEC)	J96	873	5.8	74.8
Asthma	J45	659	4.3	79.2
Viral pneumonia, unspecified	J12	435	2.9	82.0
Other respiratory disorders	J98	258	1.7	83.7
Other septicemias	A41	228	1.5	85.2
Disease caused by unspecified virus	B34	208	1.4	86.6
Unspecified bacterial infection	A49	182	1.2	87.8
Others grouped		1,850	12.2	100.0
Total		15,169	100.0	
Population < 1 year				
Acute bronchiolitis	J21	5,927	53.5	53.5
Pneumonia due to unspecified microorganism	J18	1,686	15.2	68.7
Respiratory failure, not elsewhere classified (NEC)	J96	714	6.4	75.2
Bacterial pneumonia, unspecified	J15	610	5.5	80.7
Viral pneumonia, unspecified	J12	264	2.4	83.1
Other respiratory disorders	J98	212	1.9	85.0
Other septicemias	A41	166	1.5	86.5
Unspecified bacterial infection	A49	147	1.3	87.8
Respiratory distress of newborn	P22	134	1.2	89.0
Bronchitis, unspecified as acute or chronic	J40	118	1.1	90.1
Others grouped		1,096	9.9	100.0
Total		11,074	100.0	
Population < 5 years				
Acute bronchiolitis	J21	6,582	46.2	46.2
Pneumonia due to unspecified microorganism	J18	2,538	17.8	64.1
Bacterial pneumonia, unspecified	J15	1,041	7.3	71.4
Respiratory failure, not elsewhere classified (NEC)	J96	832	5.8	77.2
Asthma	J45	544	3.8	81.1
Viral pneumonia, unspecified	J12	399	2.8	83.9
Other respiratory disorders	J98	254	1.8	85.6
Other septicemias	A41	189	1.3	87.0
Unspecified bacterial infection	A49	166	1.2	88.1
Disease caused by unspecified virus	B34	158	1.1	89.2
Others grouped		1,531	10.8	100,0
Total		14,234	100.0	
Population ≥ 60 years				
Pneumonia due to unspecified microorganisms	J18	93	24.6	24.6
Other chronic obstructive pulmonary diseases	J44	43	11.4	36.0
Heart failure	I50	32	8.5	44.4
Bacterial pneumonia, unspecified	J15	29	7.7	52.1
Disease caused by unspecified virus	B34	27	7.1	59.3
Other septicemias	A41	26	6.9	66.1
Respiratory failure, not elsewhere classified	J96	12	3.2	69.3
Viral pneumonia, unspecified	J12	11	2.9	72.2
Unspecified bacterial infection	A49	10	2.6	74.9
Chronic renal failure	N18	6	1.6	76.5
Others grouped		89	23.5	100.0
Total		378	100,0	

Table 3 summarizes the diagnostic performance of various RSV case definitions, measured by sensitivity, specificity and AUC, for the overall population and specific age groups. The inclusion of additional diagnostic codes (J21 and ARI) improves sensitivity but at the expense of specificity, particularly in younger age groups. Sensitivity shows a progressive increase from the basic RSV definition (RSV) to more inclusive definitions (RSV +  J21 and RSV + ARI) across all age categories, with the highest sensitivity observed in infants under 1 year of age. For example, the sensitivity of the RSV + ARI definition reaches 66.7% (95%CI: 65.8-67.6) in infants, compared to 61.7% (95%CI: 60.9-62.5) for children under 5 years and only 23% (95%CI: 18.9-27.6) in those over 60 years. However, this increase in sensitivity is accompanied by a reduction in specificity, particularly notable in infants (45.1%, 95%CI: 44.4-45.6), compared to children under 5 (56%, 95%CI: 55.6-56.4) and adults over 60 years (83.8%, 95%CI: 83.5-84.1). The overall diagnostic performance, reflected by the AUC, tends to be more favorable in younger populations, especially infants, but deteriorates significantly in older adults, who retain high specificity but suffer from lower sensitivity.

**Table 3:** Sensitivity, specificity, and ROC area of RSV case definitions across different age groups.

	Sensitivity		Specificity		Area under the ROC curve	
	%	95% CI	%	95% CI	(Sens+Spec.)/2	95% CI
Whole population						
RSV codes^*^	19.2	18.6-19.8	98.3	98.3-98.4	0.588	0.584-0.591
RSV codes + J21^**^	44.4	43.6-45.2	92.5	92.4-92.6	0.684	0.68-0.688
RSV codes + ARI codes^***^	59.4	58.6-60.2	75.3	75.1-75.5	0.673	0.669-0.677
Population < 1 year						
RSV codes	23.4	22.6-24.2	91.4	91-91.7	0.574	0.57-0.578
RSV codes + J21	54.2	53.3-55.2	62.3	61.8-62.9	0.583	0.577-0.588
RSV codes + ARI codes	66.7	65.8-67.6	45.1	44.4-45.6	0.559	0.554-0.564
Population < 5 years						
RSV codes	20.3	19.6-20.9	94.9	94.7-95.1	0.576	0.572-0.579
RSV codes + J21	47.1	46.2-47.9	77.1	76.7-77.4	0.621	0.616-0.625
RSV codes + ARI codes	61.7	60.9-62.5	56	55.6-56.4	0.589	0.584-0.593
Population ≥ 60 years						
RSV codes	2.1	0.9-4.1	100	99.9-100	0.51	0.503-0.518
RSV codes + J21	2.9	1.5-5.2	99.9	99.9-100	0.514	0.506-0.523
RSV codes + ARI codes	23	18.9-27.6	83.8	83.5-84.1	0.534	0.513-0.555

ICD-10 codes in use:

* RSV: J12.1, J20.5, J21.0, B97.4.

** J21: 21, J21.8, J21.9.

*** ARI: J06, J06.0, J06.8, J06.9, J11, J11.0, J11.1, J11.8, J12, J12.8, J12.9, J18, J18.0, J18.8, J18.9, J20, J20.8, J20.9, J21, J21.8, J21.9, J22, B34.9.

## Discussion

This study focused on evaluating the accuracy of RSV-specific ICD-10 coding in hospitalized patients, analyzing clinical diagnoses of patients linked to the SIVEP database where RSV was confirmed through laboratory testing. Our findings demonstrated significant variation in clinical presentations of RSV infection across age groups. In younger children, particularly those under 12 months, acute bronchiolitis was the predominant diagnosis, while older adults often presented with pneumonia, COPD, or heart failure. These variations underscore the challenges in using a uniform case definition for RSV across all age groups, as broader definitions such as RSV +  J21 (acute bronchiolitis) or RSV +  ARI (acute respiratory infection) improved sensitivity in infants but compromised specificity. Conversely, restrictive definitions, although maintaining high specificity, missed a significant number of cases, particularly in older adults.

The age-related differences in RSV presentation, as reflected through ICD-10 coding in the hospitalization database, are notable. In younger children, acute bronchiolitis dominated, with pneumonia as the second most frequent diagnosis, whereas in older adults, pneumonia was the leading diagnosis. Although most cases of pneumonia were coded as due to unspecified microorganisms, some were classified as bacterial, suggesting potential co-infections [34]. Importantly, the fact that RSV-positive patients were not recognized as such in their clinical diagnoses raises concerns about the quality of coding in hospitals. Those responsible for coding may not prioritize capturing diagnoses based on test results, as these results may only become available late in the patient’s hospitalization, if at all before discharge. This contributes to both underreporting and misclassification of RSV cases, with coding frequently reflecting clinical symptoms rather than confirmed etiologies. Even though there was an increase in testing following the COVID-19 pandemic [25], laboratory diagnostic tests are still not consistently performed for suspected RSV or other respiratory infections, even in hospital settings. This ongoing gap further complicates the accurate recording and reporting of RSV-related hospitalizations.

Diagnoses like respiratory failure, prevalent across all age groups, are nonspecific and fail to capture the underlying etiologies, reflecting what could be considered ‘garbage coding’ in this context, even though the term is typically restricted to mortality data [18]. Additionally, conditions such as asthma in young children and COPD or heart failure in older adults likely represent comorbidities exacerbated by RSV infection rather than the primary diagnosis of hospitalization [35,36]. Improving the accuracy of hospital coding requires better training for coding clerks, emphasizing the importance of recording confirmed etiologies over clinical presentations when possible. For instance, using RSV-specific codes such as J12.1 for RSV pneumonia in older adults, rather than more general respiratory codes, would enhance the accuracy of data.

Additionally, even though our selected RSV case definitions prioritized codes that could potentially mask RSV infections, it is likely that additional RSV cases are hidden within a broader array of codes. For example, an RSV infection leading to secondary bacterial pneumonia in an infant may be coded primarily as bacterial pneumonia because it may represent the most resource-intense condition and generate the highest reimbursement. While this reflects a systemic limitation in the SIH, where reimbursement priorities often overshadow accurate identification of underlying or coexisting conditions, it is difficult to quantify the extent of this influence solely through secondary data.

We anticipated that some SIVEP hospitalization records would not link to the SIH dataset, as SIH does not include private hospitalizations. However, nearly 75% of SIVEP records failed to link, despite private hospitalizations accounting for only about 25% of the total [27]. One possible explanation is that some records classified as hospitalizations in SIVEP may have involved patients treated in emergency rooms who were never formally admitted and thus not recorded in SIH. Additionally, we expected that some SIH records coded with RSV might not have been reported to SIVEP. However, we were surprised by the high proportion of 85% non-linkage, particularly since notification is mandatory. Even if patients tested negative for RSV or were not tested, they should have been reported as they met the criteria for SARS. The stringent linkage criteria employed in our study likely contributed to the low match rates observed for both issues. Minor data entry errors in any of the linkage variables could result in non-matches, as this strict approach was intentionally chosen to ensure that matched records unequivocally belonged to the same patient. Nonetheless, these findings highlight the need for improved data entry and surveillance training in the Brazilian healthcare system, alongside more effective data monitoring and follow-up to ensure compliance with compulsory notification practices.

Though other studies, like those by Cai et al. [13], suggest that coding accuracy improves slightly during peak RSV season, we chose not to investigate seasonal variation in our study due to Brazil’s vast geographic diversity. While RSV seasonality in southern Brazil generally coincides with colder months [18,19], and in central and northern regions with rainy seasons [16,17], shifting weather patterns and overlapping climatic influences across regions complicate efforts to generalize or stratify RSV trends by geographic area.

Our findings underscore the underreporting of RSV-related hospitalizations in the SIH database due to the limitations of RSV-specific ICD-10 coding. The reliance on symptomatic coding in hospitalization records, particularly when diagnostic testing is delayed or incomplete, often leads to the prioritization of clinical symptoms over confirmed etiologies. This practice contributes to the underestimation of RSV cases, complicating public health reporting and the healthcare response to respiratory infections.

As for limitations, this study relies on secondary data, which inherently carries issues of miscoding and underreporting. These challenges are not just constraints but are central to our analysis and discussion, as we aim to highlight the difficulties in accurately identifying RSV-related hospitalizations within existing databases. The linkage of SIVEP and SIH datasets, while valuable, was affected by stringent linkage criteria that likely contributed to a high proportion of unmatched records, exposing potential inaccuracies in data entry. The absence of private hospital data in SIH and the possibility that some SIVEP records classified as hospitalizations were actually emergency room visits without formal admission further complicate the analysis.

To improve the accuracy of RSV coding, several strategies should be considered. First, allowing codification to be updated after final laboratory test results are obtained could significantly reduce miscoding, ensuring that diagnoses reflect confirmed etiologies rather than clinical symptoms alone. This practice would require adjustments to current workflows and guidelines but could address a substantial proportion of misclassifications. Second, enhanced training programs for coding professionals, particularly those responsible for hospital discharge summaries, should focus on the importance of capturing confirmed diagnoses and recognizing RSV-specific codes. Third, organizational changes to prioritize the integration of laboratory diagnostics with hospital information systems would ensure that test results are readily available for coding purposes. Lastly, auditing and feedback mechanisms could be strengthened to identify and correct recurrent coding errors, further reinforcing best practices among coding staff. Together, these measures would support more accurate RSV surveillance and better inform public health interventions [37].

Improving diagnostic accuracy and coding specificity in hospital data is crucial to enhancing RSV surveillance and ensuring a more effective public health response. With the recent approval of RSV vaccines and others in development, the accurate identification of RSV cases in hospitalizations is more critical than ever. Reliable epidemiological data and long-term surveillance of RSV seasonality, supported by improved coding practices, will be essential for assessing the impact of vaccination programs and guiding public health strategies.

## References

[1] Ali A, Lopardo G, Scarpellini B, Stein RT, Ribeiro D. Systematic review on respiratory syncytial virus epidemiology in adults and the elderly in Latin America. Int J Infect Dis. 2020;90:170–80. doi: 10.1016/j.ijid.2019.10.025 31669592 PMC7110494

[2] Falsey AR, Hennessey PA, Formica MA, Cox C, Walsh EE. Respiratory syncytial virus infection in elderly and high-risk adults. N Engl J Med. 2005;352(17):1749–59. doi: 10.1056/NEJMoa043951 15858184

[3] Staadegaard L, Caini S, Wangchuk S, Thapa B, de Almeida WAF, de Carvalho FC, et al. The global epidemiology of rsv in community and hospitalized care: findings from 15 countries. Open Forum Infect Dis. 2021;8(7):ofab159. doi: 10.1093/ofid/ofab159 34337092 PMC8320297

[4] Branche AR, Falsey AR. Respiratory syncytial virus infection in older adults: an under-recognized problem. Drugs Aging. 2015;32(4):261–9. doi: 10.1007/s40266-015-0258-9 25851217

[5] Alchikh M, Conrad T, Hoppe C, Ma X, Broberg E, Penttinen P, et al. Are we missing respiratory viral infections in infants and children? Comparison of a hospital-based quality management system with standard of care. Clin Microbiol Infect. 2019;25(3):380.e9–e16. doi: 10.1016/j.cmi.2018.05.023 29906596

[6] Feemster KA, Leckerman KH, Middleton M, Zerr DM, Elward AM, Newland JG, et al. Use of Administrative Data for the Identificationof Laboratory-Confirmed Influenza Infection: The Validity ofInfluenza-Specific ICD-9 Codes. J Pediatric Infect Dis Soc. 2013;2(1):63–6. doi: 10.1093/jpids/pis052 26619444

[7] Amodio E, Tramuto F, Costantino C, Restivo V, Maida C, Calamusa G, et al. Diagnosis of influenza: only a problem of coding?. Med Princ Pract. 2014;23(6):568–73. doi: 10.1159/000364780 25059566 PMC5586933

[8] Moore HC, Lehmann D, de Klerk N, Smith DW, Richmond PC, Keil AD, et al. How Accurate Are International Classification of Diseases-10 Diagnosis Codes in Detecting Influenza and Pertussis Hospitalizations in Children?. J Pediatric Infect Dis Soc. 2014;3(3):255–60. doi: 10.1093/jpids/pit036 26625389

[9] Köpke K, Prahm K, Buda S, Haas W. Evaluation of an ICD-10-based electronic surveillance of acute respiratory infections (SEEDARI) in Germany. Bundesgesundheitsblatt Gesundheitsforschung Gesundheitsschutz. 2016;59(11):1484–91. doi: 10.1007/s00103-016-2454-0 27738704

[10] Antoon JW, Stopczynski T, Amarin JZ, Stewart LS, Boom JA, Sahni LC, et al. Accuracy of Influenza ICD-10 Diagnosis Codes in Identifying Influenza Illness in Children. JAMA Netw Open. 2024;7(4):e248255. doi: 10.1001/jamanetworkopen.2024.8255 38656577 PMC11043895

[11] Benack K, Nyandege A, Nonnenmacher E, Jan S, Setoguchi S, Gerhard T, et al. Validity of ICD-10-based algorithms to identify patients with influenza in inpatient and outpatient settings. Pharmacoepidemiol Drug Saf. 2024;33(4):e5788. doi: 10.1002/pds.5788 38556924 PMC11022168

[12] Pisesky A, Benchimol EI, Wong CA, Hui C, Crowe M, Belair M-A, et al. Incidence of Hospitalization for Respiratory Syncytial Virus Infection amongst Children in Ontario, Canada: A Population-Based Study Using Validated Health Administrative Data. PLoS One. 2016;11(3):e0150416. doi: 10.1371/journal.pone.0150416 26958849 PMC4784925

[13] Cai W, Tolksdorf K, Hirve S, Schuler E, Zhang W, Haas W, et al. Evaluation of using ICD-10 code data for respiratory syncytial virus surveillance. Influenza Other Respir Viruses. 2020;14(6):630–7. doi: 10.1111/irv.12665 31206246 PMC7578302

[14] Egeskov-Cavling AM, Johannesen CK, Lindegaard B, Fischer TK, PROMISE Investigators. Underreporting and Misclassification of Respiratory Syncytial Virus-Coded Hospitalization Among Adults in Denmark Between 2015-2016 and 2017-2018. J Infect Dis. 2024;229(Supplement_1):S78–83. doi: 10.1093/infdis/jiad415 37747825

[15] Freitas FT de M. Sentinel surveillance of influenza and other respiratory viruses, Brazil, 2000-2010. Braz J Infect Dis. 2013;17(1):62–8. doi: 10.1016/j.bjid.2012.09.001 23287541 PMC9427376

[16] Moura FEA, Nunes IFS, Silva GB Jr, Siqueira MM. Respiratory syncytial virus infections in northeastern Brazil: seasonal trends and general aspects. Am J Trop Med Hyg. 2006;74(1):165–7. doi: 10.4269/ajtmh.2006.74.165 16407363

[17] Santos R-KO, Borges IC, Souza ML, Bouzas ML, Nascimento-Carvalho CM. Seasonality of distinct respiratory viruses in a tropical city: implications for prophylaxis. Trop Med Int Health. 2021;26(6):672–9. doi: 10.1111/tmi.13571 33666303

[18] Tumba K, Comaru T, Machado C, Ribeiro M, Pinto LA. Temporal trend of hospitalizations for acute bronchiolitis in infants under one year of age in Brazil between 2008 and 2015. Rev Paul Pediatr. 2019;38:e2018120. doi: 10.1590/1984-0462/2020/38/2018120 31778406 PMC6909255

[19] Vieira SE, Stewien KE, Queiroz DA, Durigon EL, Török TJ, Anderson LJ, et al. Clinical patterns and seasonal trends in respiratory syncytial virus hospitalizations in São Paulo, Brazil. Rev Inst Med Trop Sao Paulo. 2001;43(3):125–31. doi: 10.1590/s0036-46652001000300002 11452319

[20] Ciapponi A, Palermo MC, Sandoval MM, Baumeister E, Ruvinsky S, Ulloa-Gutierrez R, et al. Respiratory syncytial virus disease burden in children and adults from Latin America: a systematic review and meta-analysis. Front Public Health. 2024;12:1377968. doi: 10.3389/fpubh.2024.1377968 39478747 PMC11521816

[21] Roteli-Martins CM, Ballalai I de AM, Kfouri R de Á, Fialho SCAV. Respiratory syncytial virus: impact of the disease and preventive strategies in pregnant women and older adults: Number 6 - 2024. Rev Bras Ginecol Obstet. 2024;46:e-FPS06. doi: 10.61622/rbgo/2024FPS06 39381334 PMC11460417

[22] Lipp MA, Empey KM. Recent advances in the prevention of respiratory syncytial virus in pediatrics. Curr Opin Pediatr. 2024;36(2):182–9. doi: 10.1097/MOP.0000000000001336 38299987 PMC11189640

[23] Mazur NI, Terstappen J, Baral R, Bardají A, Beutels P, Buchholz UJ, et al. Respiratory syncytial virus prevention within reach: the vaccine and monoclonal antibody landscape. Lancet Infect Dis. 2023;23(1):e2–21. doi: 10.1016/S1473-3099(22)00291-2 35952703 PMC9896921

[24] Venkatesan P. Advances in preventing RSV in children. Lancet Microbe. 2024;5(5):e421. doi: 10.1016/S2666-5247(24)00043-0 38437849

[25] Almeida GB de, Grott RMT, Fortaleza CMCB, Ferreira CP, Vilche TN, Guimarãe RB, et al. Aumento da capacidade para o diagnóstico molecular da covid‐19 no brasil ao longo de 100 dias de epidemia. The Brazilian Journal of Infectious Diseases. 2021;25 101088. doi: 10.1016/j.bjid.2020.101088

[26] Duarte E, Eble LJ, Garcia LP. 30 years of the Brazilian National Health System. Epidemiol Serv Saude. 2018;27(1):e00100018. doi: 10.5123/S1679-49742018000100018 29590234

[27] Souza Júnior PRB de, Szwarcwald CL, Damacena GN, Stopa SR, Vieira MLFP, Almeida W da S de, et al. Health insurance coverage in Brazil: analyzing data from the National Health Survey, 2013 and 2019. Cien Saude Colet. 2021;26(suppl 1):2529–41. doi: 10.1590/1413-81232021266.1.43532020 34133632

[28] Mussi CC, Luz R, Damázio D da R, Santos EMD, Sun V, Porto BS da S, et al. The Large-Scale Implementation of a Health Information System in Brazilian University Hospitals: Process and Outcomes. Int J Environ Res Public Health. 2023;20(21):6971. doi: 10.3390/ijerph20216971 37947529 PMC10650123

[29] Cerqueira DRC, Alves PP, Coelho DSC, Reis MVM, Lima AS. Uma análise da base de dados do Sistema de Informação Hospitalar entre 2001 e 2018: dicionário dinâmico, disponibilidade dos dados e aspectos metodológicos para a produção de indicadores sobre violência. IPEA; 2019. Available from: https://www.ipea.gov.br/atlasviolencia/arquivos/artigos/9058-sistemahospitalar.pdf.

[30] Brasil. Ministério da Saúde. Secretaria de Atenção à Saúde. SIH – Sistema de Informação Hospitalar do SUS: Manual Técnico Operacional do Sistema; 2017. Available from: http://sihd.datasus.gov.br/documentos/documentos_sihd2.php.

[31] Cantarino L, Merchan-Hamann E. Influenza in Brazil: surveillance pathways. J Infect Dev Ctries. 2016;10(1):13–23. doi: 10.3855/jidc.7135 26829533

[32] Secretaria de Saúde do Estado da Bahia. Guia rápido Sivep-Gripe; 2021. Available from: https://docs.bvsalud.org/biblioref/2021/03/1147534/guia-rapido-sivep-gripe_agosto-2020.pdf

[33] Brasil. Ministério da Saúde. Secretaria de Vigilância em Saúde e Ambiente. Departamento de Análise Epidemiológica e Vigilância de Doenças Não Transmissíveis. [Guia de vigilância integrada da Covid-19, Influenza e outros vírus respiratórios de importância em saúde pública]; 2024. Available from: http://bvsms.saude.gov.br/bvs/publicacoes/guia_vigilancia_integrada_covid19_Influenza.pdf.

[34] Pacheco GA, Gálvez NMS, Soto JA, Andrade CA, Kalergis AM. Bacterial and Viral Coinfections with the Human Respiratory Syncytial Virus. Microorganisms. 2021;9(6):1293. doi: 10.3390/microorganisms9061293 34199284 PMC8231868

[35] Chan CY, Low JG, Wyone W, Oon LL, Tan BH. Survey of Respiratory Virus in Patients Hospitalised for Acute Exacerbations of Heart Failure - A Prospective Observational Study. Ann Acad Med Singap. 2018;47(11):445–50. doi: 10.47102/annals-acadmedsg.v47n11p445 30578423

[36] Hewitt R, Farne H, Ritchie A, Luke E, Johnston SL, Mallia P. The role of viral infections in exacerbations of chronic obstructive pulmonary disease and asthma. Ther Adv Respir Dis. 2016;10(2):158–74. doi: 10.1177/1753465815618113 26611907 PMC5933560

[37] Martinón-Torres F, Navarro-Alonso JA, Garcés-Sánchez M, Soriano-Arandes A. The Path Towards Effective Respiratory Syncytial Virus Immunization Policies: Recommended Actions. Arch Bronconeumol. 2023;59(9):581–8. doi: 10.1016/j.arbres.2023.06.006 37414639

